# Performance of CAC-prob in predicting coronary artery calcium score: an external validation study in a high-CAC burden population

**DOI:** 10.1186/s12911-025-03128-y

**Published:** 2025-08-04

**Authors:** Pakpoom Wongyikul, Phichayut Phinyo, Pannipa Suwannasom, Apichat Tantraworasin, Chanikan Srikuenkaew, Pichyapa Jira, Kempiya Pornpipatsakul, Arachaporn Ngachuea, Phansa Chanthanom, Kanogphol Prayongkul, Warisara Chavalitjiraphan, Thitirat Rattananalin, Surasak Saokaew

**Affiliations:** 1 Department of Biomedical Informatics and Clinical Epidemiology (BioCE), Faculty of Medicine, Chiang Mai University, Chiang Mai, 50200 Thailand; 2 Center for Clinical Epidemiology and Clinical Statistics, Faculty of Medicine, Chiang Mai University, Chiang Mai, 50200 Thailand; 3 Department of Biomedical Informatics and Clinical Epidemiology (BioCE), Faculty of Medicine, Chiang Mai University, Chiang Mai, 50200 Thailand; 4 Center of Multidisciplinary Technology for Advanced Medicine (CMUTEAM), Faculty of Medicine, Chiang Mai University, Chiang Mai, 50200 Thailand; 5 Division of Cardiology, Department of Internal Medicine, Faculty of Medicine, Chiang Mai University, Chiang Mai, 50200 Thailand; 6 General Thoracic Unit, Department of Surgery, Faculty of Medicine, Chiang Mai University Hospital, Chiang Mai, 50200 Thailand; 7 Faculty of Medicine, Chiang Mai University, Chiang Mai, 50200 Thailand; 8 Division of Social and Administrative Pharmacy, Department of Pharmaceutical Care, School of Pharmaceutical Sciences, University of Phayao, Phayao, 56000 Thailand; 9 Unit of Excellence on Clinical Outcomes Research and Integration (UNICORN), School of Pharmaceutical Sciences, University of Phayao, Phayao, 56000 Thailand; 10 Center of Health Outcomes Research and Therapeutic Safety (Cohorts), School of Pharmaceutical Sciences, University of Phayao, Phayao, 56000 Thailand; 11 Center of Excellence in Bioactive Resources for Innovative Clinical Applications, Chulalongkorn University, Bangkok, 10330 Thailand

**Keywords:** Clinical prediction model, Coronary Artery Calcium, Outpatients

## Abstract

**Background:**

Although CAC screening is gaining recognition in developing countries such as Thailand, official guidelines for using the CAC score in cardiovascular risk assessment remain lacking. This study aims to externally validate CAC-prob, a recently developed prediction model that can estimate the probability of CAC > 0 and CAC ≥ 100, to confirm its robustness.

**Method:**

This study externally validated the CAC-prob model using retrospective data from a tertiary care centre in northern Thailand. Patients who underwent CAC screening between 2019 and 2022 were included. CAC-prob consists of two models: one predicting the probability of CAC > 0 (Model 1) and another predicting the probability of CAC ≥ 100 (Model 2). Model performance was assessed in terms of discrimination (Ordinal C-index), calibration slope, and diagnostic indices for each model.

**Results:**

A total of 329 patients were included. The patient characteristics observed in this study indicated a higher prevalence of DM, hypertension, dyslipidaemia, CKD, and CAC ≥ 100 compared to the development study. The ordinal C-index derived from the validation study showed a slight decline (0.78). The calibration slope for Model 1 and Model 2 was 1.28 (95% CI 0.95–1.63) and 1.06 (95% CI 0.78–1.36), respectively. In Model 1, CAC-prob demonstrated comparable diagnostic performance. However, in Model 2, it showed slightly better performance, with significantly improved sensitivity compared to the development study.

**Conclusion:**

This external validation study confirms the predictive performance of CAC-prob in Northern Thai patients. The findings support the integration of CAC-prob into routine clinical practice to aid physicians in making recommendations for CAC screening.

**Supplementary Information:**

The online version contains supplementary material available at 10.1186/s12911-025-03128-y.

## Introduction

The Coronary Artery Calcium (CAC) score, a non-invasive imaging method for evaluating coronary artery calcification, was introduced in the late 1990s [1]. The score provides a precise quantification of calcified atherosclerotic plaque within the coronary arteries and has demonstrated a strong association with major adverse cardiovascular events (MACE) [2, 3]. Research and guidelines over the past decades have consistently validated the CAC score as a reliable tool for cardiovascular risk stratification [4–7]. It outperforms conventional risk scores in predictive accuracy and further enhances their utility in assessing atherosclerotic cardiovascular disease (ASCVD) risk when combined [4, 8].

The increased availability of CT scanners has led to wider use of CAC screening in many developing countries, including Thailand. However, evidence on its effectiveness in Thai patients remains limited, delaying the development of official guidelines for using CAC scores in cardiovascular risk assessment or setting cut-off points for starting statin therapy [9]. Early studies suggest that CAC scores could reliably predict MACE, and using absolute CAC score classifications may be more effective for risk stratification in the Thai clinical population [10, 11]. Importantly, CAC is meant to support ASCVD risk assessment, not replace it. Current Thai official guidelines for ASCVD prevention (2024) do not include the supplemental use of CAC scores for risk stratification. In the absence of national guidelines, CAC screening could be exploited commercially, driving up healthcare costs and leading to unnecessary tests and expenses [12, 13].

Recently, CAC-prob, a clinical prediction model that calculates the probability of CAC > 0 and CAC ≥ 100 in individual patients, was developed. It utilises four routine clinical predictors: age, sex, presence of hypertension or diabetes mellitus (DM), and low levels of high-density lipoprotein cholesterol (HDL-C). CAC-prob has shown promising predictive performance and has the potential to aid physicians in recommending CAC screening while ensuring cost-effectiveness [14]. Since prediction models are designed to be applied to new individuals, their true utility depends on performance beyond the development cohort. Therefore, all prediction models should be validated before clinical implementation [15]. This study aims to externally validate CAC-prob to ensure the robustness of its performance in a cardiology outpatient unit.

## Methods

### The CAC-prob model

**Fig. 1:**
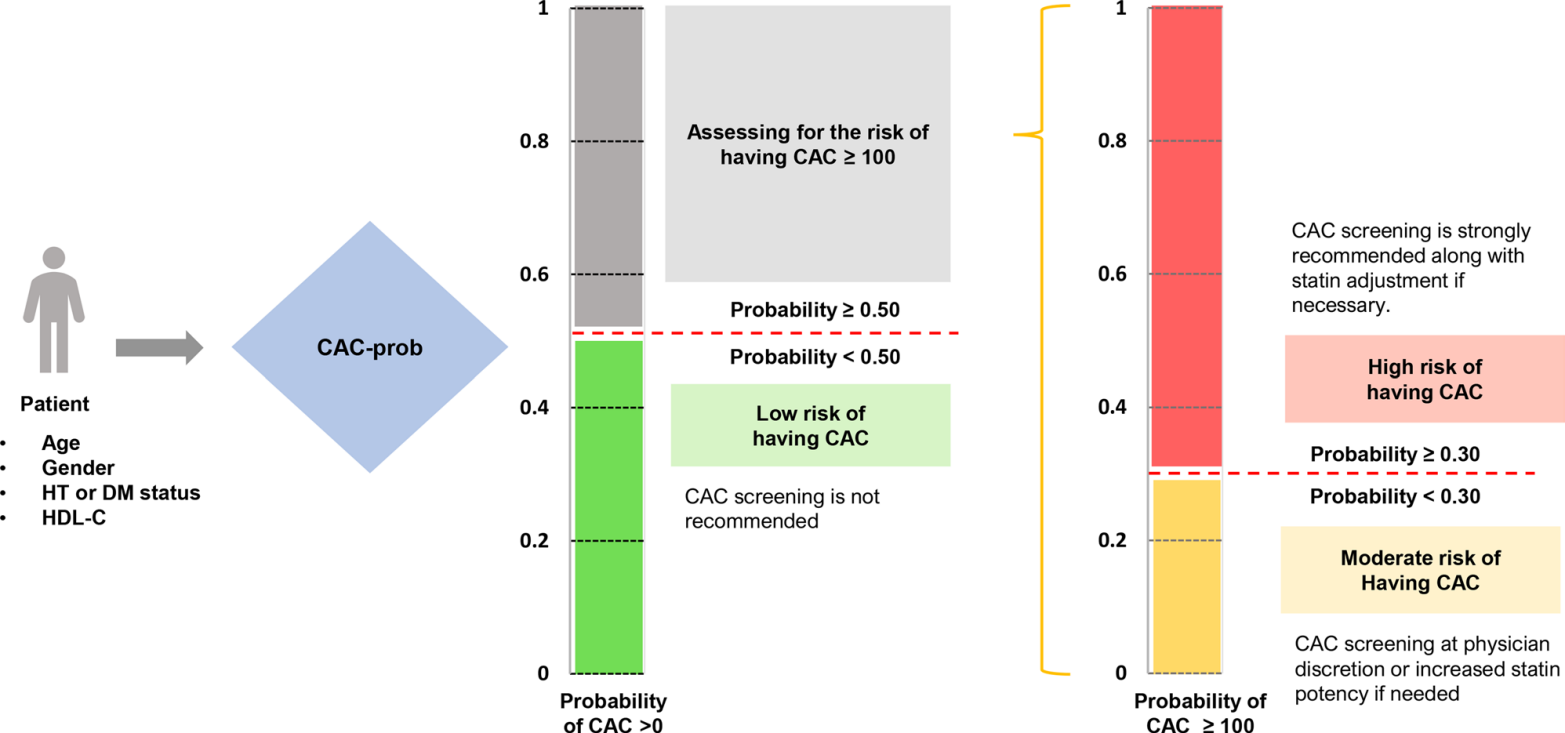
Recommendation algorithm of the CAC-prob model. After inputting age, gender, HT/DM status, and HDL-C levels, patients are assessed for CAC > 0 probability. Those with < 0.50 probability are classified as low risk, and CAC screening is not recommended. For patients with ≥ 0.50 probability, CAC > 100 probability is evaluated. A probability < 0.30 categorises them as moderate risk, with screening at physician discretion or increased statin potency if needed. A probability ≥ 0.30 indicates high risk, where CAC screening is strongly recommended along with statin adjustment if necessary. Abbreviations: HT, hypertension; DM, diabetes mellitus; HDL-C, high-density lipoprotein cholesterol; CAC, coronary artery calcium

CAC-prob was derived from data collected between 2012 and 2018 at Maharaj Nakorn Chiang Mai Hospital using four key predictors: age, sex, presence of hypertension or DM, and low HDL-C (<40 mg/dL in males, < 50 mg/dL in females). The outcome categories were CAC scores of 0, 1–99, and ≥ 100. Consequently, CAC-prob comprises two models: one predicting the probability of CAC > 0 (Model 1) and another predicting the probability of CAC ≥ 100 (Model 2), with corresponding cut-off points of 0.50 and 0.30, respectively. Details of the recommendation algorithm are shown in Fig. 1.

### Study design

This study externally validated the CAC-prob model using retrospective data from Maharaj Nakorn Chiang Mai Hospital between January 2019 and December 2022. Ethical approval for this study was granted by the Institutional Review Board and Ethics Committee of the Faculty of Medicine, Chiang Mai University (HOS2566–0097). The requirement for informed consent was waived. This study was conducted in accordance with the principles of the Declaration of Helsinki. The study was reported in accordance with the Transparent Reporting of a Multivariable Prediction Model for Individual Prognosis or Diagnosis (TRIPOD) guidelines [16].

### Patients and data collection

All patients who underwent CAC screening were included. Patients without an official CAC score report or with a history of MACE prior to CAC screening were excluded. For consistency, all data extraction followed the framework of the original study [14]. Demographic data, clinical characteristics, and laboratory parameters were collected on the date of CAC screening or, if unavailable, within 30 days of the screening date. Missing data were handled under the assumption of missing at random (MAR), which is a more plausible assumption than complete case analysis, given the observed covariates in our study [17]. We employed multiple imputation with chained equation (MICE) using a fully conditional specification (FCS) approach [18].

### Coronary artery calcium score

The CAC score was measured using a semi-automatic, non-contrast, ECG-gated scan on a 192-slice Dual Source CT scanner (Somatom Force, Siemens). The scan was performed in systolic ECG-triggered sequential shuttle mode with a delay of 280–320 milliseconds. In all patients, the most cranial section was positioned at 65% of the R–R interval, with a slice thickness of 3 mm. The extent of CAC was quantified by certified radiologists using the Agatston scoring method [19].

### Sample size estimation

Sample size criteria for the external validation of clinical prediction models have been established for binary and continuous outcomes [20, 21], but not yet for multinomial prediction models. We adapted these criteria by calculating the minimum required sample size according to Criteria I and III for each model (one-vs-one) and selecting the larger value [22]. Criteria I and III required the outcome prevalence and expected c-statistic for each model, as reported in the original study. The minimum sample sizes required were 229 for Model 1, including 92 patients with a CAC score of 0, and 319 for Model 2, including 224 patients with a CAC score ≥ 100.

### External validation

We followed the validation and updating steps for clinical models with polytomous outcomes proposed by Gehringer et al. [22]. First, patient characteristics were compared between the validation and development studies. Categorical variables were summarised as frequencies and percentages, while numerical variables were described using the mean and standard deviation (SD) for normally distributed data or the median and interquartile range (IQR) for non-normally distributed data. The standardised difference (Std diff) was used to assess the equivalence of baseline characteristics between the two studies. An Std diff greater than ± 10% indicated a significant baseline difference. The predictors in the model were also assessed for their association with the outcome.

Second, the prediction model was validated by fitting the reported linear predictor (LP) to the partial proportional odds model. Model performance was assessed in terms of discrimination, calibration, and diagnostic indices for each model. Discriminative performance was evaluated using the average dichotomous C-index, generalised C-index, and ordinal C-index (ORC), with a C-index between 0.70 and 0.80 considered acceptable [23]. The ORC assessed the overall performance of CAC-prob by accounting for the ordinal nature of the outcome, while the other methods averaged discriminative ability across each pair of outcomes. Calibration plots were used to assess the agreement between observed and predicted probabilities across the prediction range. Calibration slopes were also calculated, where a slope of 1.0 indicates perfect calibration. Slopes below 1.0 suggest underestimated risk, while slopes above 1.0 indicate overestimated risk or an underfitted model [24]. Diagnostic indices, including sensitivity, specificity, positive predictive value (PPV), negative predictive value (NPV), and classification metrics, were evaluated at each cut-off point.

Third, if significant model deterioration was observed, additional model updates, such as recalibration, model refitting, or re-evaluation of cut-off points, were performed. All statistical analyses were conducted using Stata 17 (StataCorp, College Station, TX, USA) or R statistical software version 4.1.2 (R Project for Statistical Computing). P values less than 0.05 were considered statistically significant.

## Results

### Comparison of patient characteristics

**Fig. 2:**
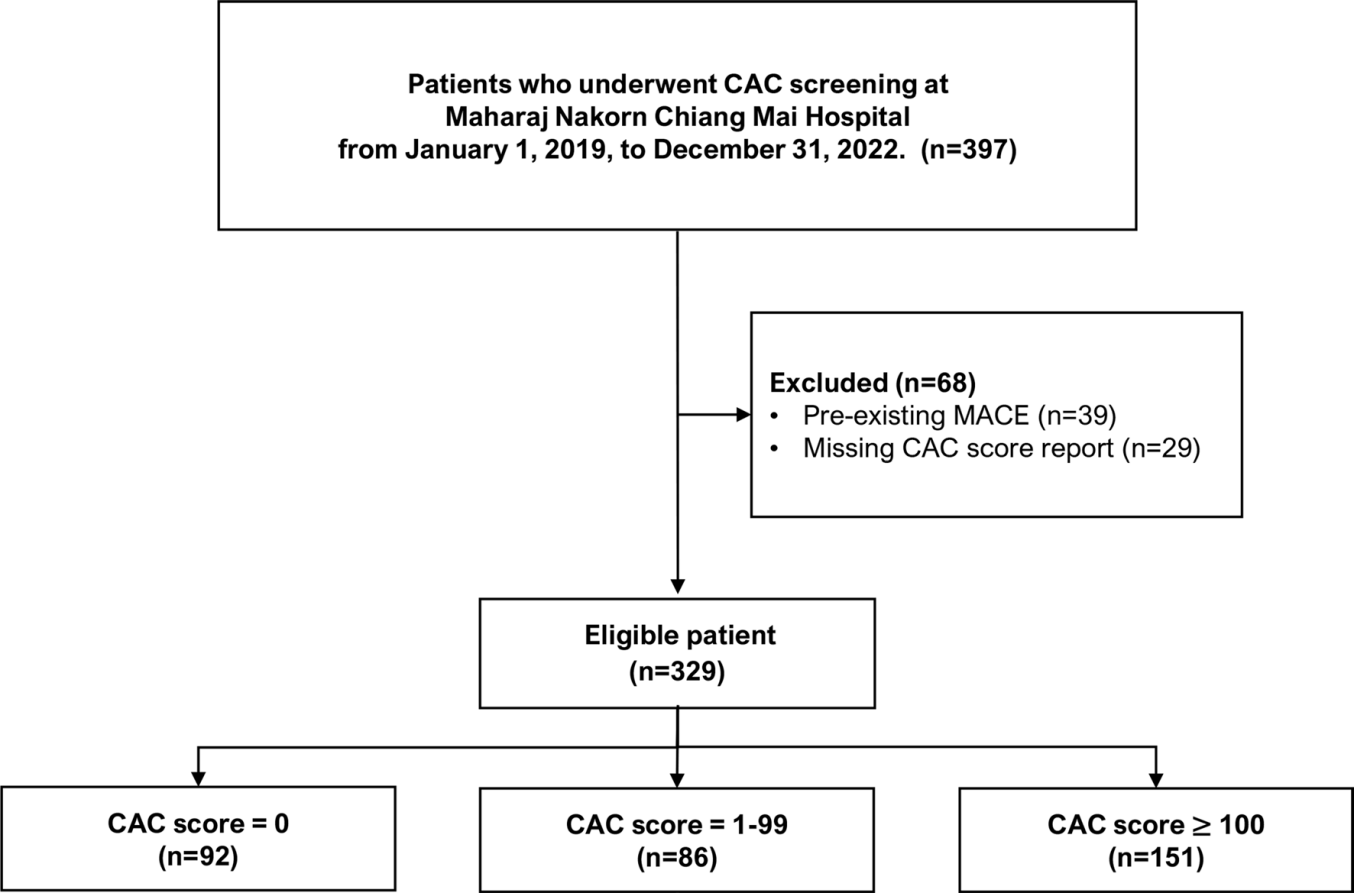
Study flow diagram. Abbreviations: CAC, coronary artery calcium; MACE, major adverse cardiovascular event

Between January 2019 and December 2022, 397 patients underwent CAC screening. Sixty-eight patients were excluded—39 due to pre-existing MACE and 29 due to missing official CAC score reports (Fig. 2)—resulting in 329 eligible patients. The mean age of patients in the validation dataset was slightly higher than in the development dataset. The proportions of patients who were current smokers or had DM, hypertension, dyslipidaemia, or chronic kidney disease (CKD) were higher than in the development study. Several factors, including HDL-C, LDL-C, triglycerides, and the prevalence of patients using lipid-lowering drugs, showed significant differences with an Std diffgreater than 10%. A higher prevalence of CAC ≥ 100 (45.9% vs 31%) was also observed in the validation study. Approximately 40% of all patients experienced symptomatic chest pain in both studies, with a similar distribution across CAC subcategories (Supplementary Table S1). Notably, 97.6% of patients underwent CAC screening for ASCVD risk assessment, a significant increase from 83.9% in the development cohort. Additional details on patient characteristics and missing data are shown in Table 1. Characteristics for all CAC subcategories in the validation study are reported in Supplementary Table S1.

**Table 1:** Comparison of clinical characteristics of the included patients between development and validation study

Prognostic factors	Missing n (%)	Validation study (n = 329) n (%)	Development study (n = 360) n (%)	Std diff
Age (years) mean, SD	2 (0.6)	64.5 ± 11.0	61.4 ± 10.6	17.4
Male	0 (0)	161 (48.9)	167 (46.4)	10.3
Smoking status	53 (16.1)			16.1
never smoking		243 (73.9)	233 (64.7)	
former smoking		22 (6.7)	14 (3.9)	
current smoking		11 (3.3)	8 (2.2)	
DM	31 (9.4)	70 (21.3)	50 (13.9)	6.2
Hypertension	27 (8.2)	205 (62.3)	179 (49.7)	9.1
Antihypertensive drug	30 (9.1)	162 (49.2)	147 (40.8)	−6.9
HDL-C(mg/dL) mean, SD	117 (35.6)	56.0 ± 18.9	53.8 ± 14.8	11.5
LDL-C(mg/dL) mean, SD	107 (32.5)	113.3 ± 44.7	117.1 ± 38.1	−14.9
Triglyceride median, IQR	115 (35.0)	101.5 (79, 128)	109 (81, 147)	−32.5
Dyslipidaemia	24 (7.3)	218 (66.3)	178 (49.4)	5.4
Lipid lowering drug	31 (9.4)	140 (42.6)	132 (36.7)	−10.4
eGFR mean, SD	59 (17.9)	75.1 ± 22.14	80.1 ± 19.3	−7.5
Chronic kidney disease	29 (8.8)	118 (35.9)	32 (8.9)	51.1
Symptomatic chest pain	29 (8.8)	116 (41.0)	140 (38.9)	−8.9
CAC screening for ASCVD risk assessment	8 (2.4)	272 (97.6)	302 (83.9)	−20.2
CAC category				14.6
CAC = 0		92 (28.0)	136 (37.8)	
CAC 1–99		86 (26.1)	113 (31.4)	
CAC ≥ 100		151 (45.9)	111 (30.8)	

**Abbreviations**: ASCVD, atherosclerotic cardiovascular disease; CAC, coronary artery calcium; DM, diabetes mellitus; eGFR, estimated glomerular infiltration rate; HDL-C, high-density lipoprotein cholesterol; IQR, interquartile range; LDL-C, Low-density lipoprotein cholesterol; SD, standard deviation; Std diff, standardised mean difference.

### Predictor-outcome associations

All predictors demonstrated slightly higher adjusted odds ratios compared to the development study and remained statistically significant (Table 2).

**Table 2:** The predictor-outcome associations in the validation and the development study

Predictors	Validation study (n = 329)			Development study (n = 360)		
	OR (95% CI)	Beta coefficient	P value	OR (95% CI)	Beta coefficient	P value
Age	1.09 (1.07–1.12)	0.09	<0.001	1.08 (1.06–1.11)	0.08	<0.001
Male	3.33 (2.10–5.28)	1.20	<0.001	2.85 (1.86–4.35)	1.05	<0.001
Hypertension or DM	2.09 (1.21–3.61)	0.74	0.008	1.78 (1.09–2.92)	0.58	0.021
Low HDL-C	2.84 (1.21–6.70)	1.04	0.017	2.31 (1.32–4.05)	0.84	0.003
Constant at cut > 0		−5.85			−5.32	
Constant cut ≥ 100		−7.18			−6.92	

**Abbreviations**: CAC, coronary artery calcium; CI, confidence interval; DM, diabetes mellitus; HDL-C, high-density lipoprotein cholesterol; OR, multivariable odds ratio

### External model performance

**Fig. 3:**
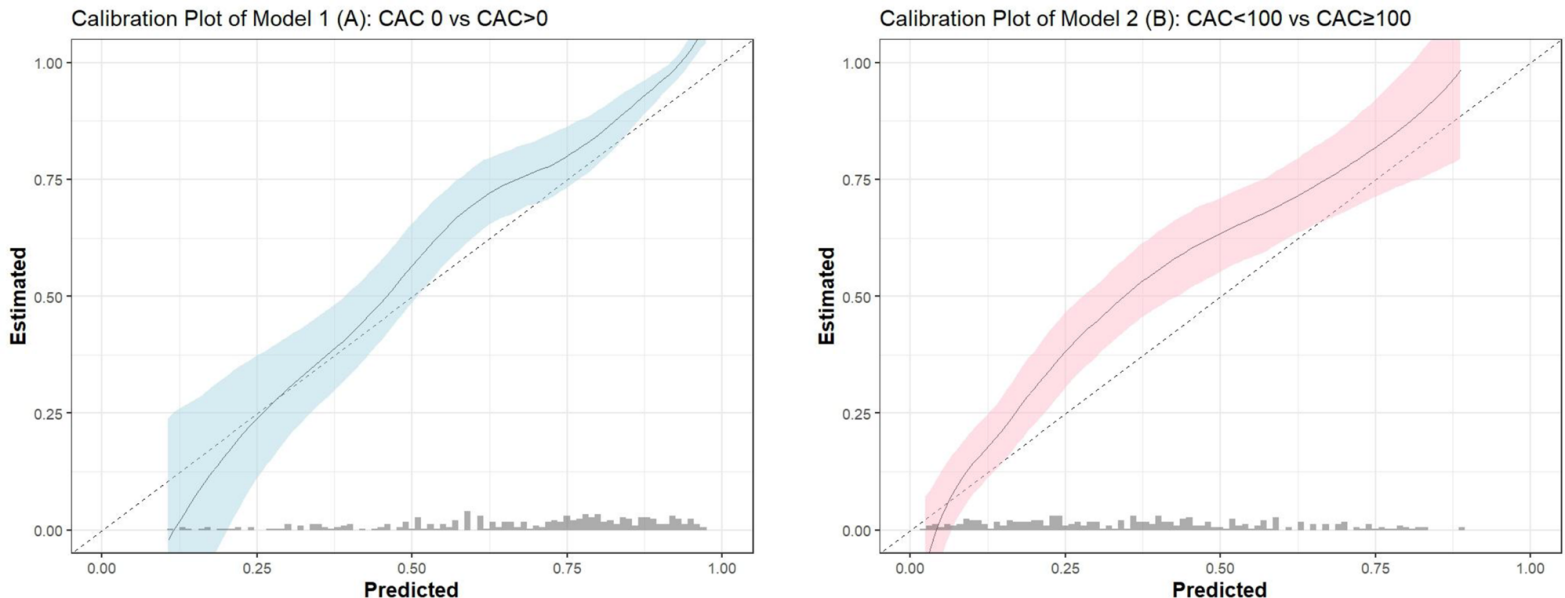
Calibration plots of the CAC-prob predictions. **A**-**B** illustrate the calibration plot for the agreement between predicted and observed probability of Model 1 (CAC > 0 vs CAC 0) and Model 2 (CAC ≥ 100 vs CAC < 100), respectively. Abbreviation: CAC, coronary artery calcium

The ORC derived from the validation dataset exhibited a slight decline (0.78). However, the average C-index and generalised C-index, which demonstrated the discriminative performance of each model, showed better performance (Table 3). Figure 3 presents the visual assessment of calibration, with both models indicating modest underfitting, with slopes of 1.28 (95% CI 0.95–1.63) and 1.06 (95% CI 0.78–1.36), respectively.

**Table 3:** Discriminative ability of the CAC-prob model based on three concordance indices: Generalized C-index, Average C-index, Ordinal C-index

Discriminative ability	Dataset	Average Value	0 vs > 0	<100 vs ≥ 100	0 vs 1-99	0 vs ≥ 100	1-99 vs ≥ 100
Generalized C-index	Development	0.73			0.75	0.83	0.60
	Validation	0.76			0.74	0.86	0.68
Average C-index	Development	0.76	0.79	0.73			
	Validation	0.80	0.82	0.77			
Ordinal C-index	Development	0.81					
	Validation	0.78					

In Model 1, CAC-prob demonstrated comparable diagnostic performance between the validation and development studies. However, in Model 2, it showed slightly better performance, with significantly improved sensitivity (validation: 78.8%, 95% CI: 71.4–85.0 vs development: 61.9%, 95% CI: 52.3–70.9) (Table 4). The overall correct classification rate was also slightly higher in Model 1 (81% vs 78%) but markedly higher in Model 2 (65% vs 56%) (Supplementary Tables S2–S3). This improvement is due to CAC-prob’s higher accuracy in classifying high-risk CAC patients while maintaining consistent classification rates in lower-risk patients compared to the development study (Supplementary Figure S1).

**Table 4:** Diagnostic indices for each cut point within model

Cut points	Dataset	Sensitivity (95% CI)	Specificity (95% CI)	PPV (95% CI)	NPV (95% CI)
Cut point for CAC screening is recommended (Model 1 cut point)					
0.50	Developed	88.4 (83.2–92.4)	57.4 (47.5–66.9)	79.9 (74.1–84.9)	72.1 (61.4–81.2)
	Validated	90.3 (85.8–93.7)	55.4 (44.7–65.8)	83.9 (78.8–88.2)	68.9 (57.1–79.2)
Cut point for CAC screening is strongly recommended (Model 2 cut point)					
0.30	Developed	61.9 (52.3–70.9)	64.4 (57.3–71.0)	49.3 (40.8–57.8)	75.1 (68.0–81.4)
	Validated	78.8 (71.4–85.0)	64.0 (56.5–71.1)	65.0 (57.6–71.9)	78.1 (70.5–84.5)

Abbreviations: CAC, coronary artery calcium; CI, confidence interval; NPV, Negative predictive value; PPV, Positive predictive value

## Discussion

This study is the first external validation of the CAC-prob model in Thai patients without prior MACE. CAC-prob demonstrated robust discriminative performance with minimal miscalibration. Additionally, the classification metrics indicated higher overall accuracy, particularly in correctly classifying patients with CAC ≥ 100. These findings support the integration of CAC-prob into routine clinical practice to assist physicians in making recommendations for CAC screening.

This study was originally designed as a temporal validation, sampling from the same clinical setting at a later time point. However, the observed patient characteristics revealed a higher-risk case mix, with a significantly increased prevalence of comorbidities, including DM, hypertension, dyslipidaemia, and CKD, as well as a greater prevalence of patients with CAC ≥ 100 compared to the development sample. Consequently, this study also provides elements of domain validation. We found that the original magnitudes of association for predictors such as male sex, hypertension or diabetes, and low HDL-C were smaller than their true magnitudes in the validation cohort (Table 2). Consequently, the model’s original linear predictor likely underestimates actual risk, resulting in model underfitting. The model showed an improvement in correctly classifying patients with CAC ≥ 100 compared to the original. It could be argued that this high accuracy is due to the increased prevalence of CAC ≥ 100 in this study. However, the model maintained consistent performance in sensitivity and specificity, which are minimally affected by outcome prevalence [25]. Therefore, model updating may not be necessary in this study.

Recent studies have shown a dramatic global increase in CAC screening [26]. Consistent with our findings, nearly 100% of patients in our sample underwent CAC screening for ASCVD risk assessment, compared to 84% of patients between 2012 and 2018 [14]. With very few exceptions, the CAC score is a well-established marker of coronary atherosclerosis. While the CAC score is strongly correlated with coronary artery plaque volume measured at autopsy and serves as a surrogate for overall coronary plaque burden [27], it does not indicate luminal stenosis or obstruction [13, 28]. Asymptomatic individuals were frequently referred for stress testing following CAC screening, which often led to coronary angiography and other invasive interventions [12, 13]. Potential harms from CAC screening include radiation exposure, incidental findings, and misdiagnosis [29, 30]. Furthermore, a substantial number of patients with no detectable CAC scores have reported experiencing symptomatic chest pain [14, 31]. While the absence of coronary calcification may indicate a lower likelihood of disease, it does not definitively exclude the presence of obstructive stenosis or the need for revascularisation in patients with a sufficiently high suspicion of coronary artery disease to warrant coronary angiography [31, 32].

CAC screening is valuable only if it leads to the implementation of effective prevention strategies to reduce future coronary events, as outlined in clinical guidelines [12]. In our setting, where guidelines for CAC screening are unavailable, CAC-prob could play an important role as a triage tool by advising against CAC screening for patients unlikely to benefit, thereby preventing unnecessary costs and downstream testing. The recommendations provided by CAC-prob also extend to patients presenting with symptomatic chest pain, as some symptomatic patients are likely to have low CAC scores. In such cases, other diagnostic tests might offer a more straightforward benefit. In addition, it helps identify patients for whom CAC screening provides valuable information to initiate or intensify treatment. In this study, 21% of participants had diabetes mellitus and would typically be started on statins due to their high risk. However, a high predicted CAC ≥ threshold by CAC-prob could prompt formal CAC measurement, thereby providing an opportunity to optimise and intensify their regimen [33]. The cut-off point is adjustable depending on contextual factors such as the affordability of CAC screening and the distribution of CAC scores in the population. The CAC-prob calculator is available in the supplementary file.

### Strength and limitation

A primary strength of our study is its extension beyond temporal to domain validation, demonstrating robust predictive performance in higher-risk ASCVD patients. Additionally, the validation process was comprehensively conducted in adhering to the guideline, ensuring both methodological rigour and transparency. However, some limitations should be acknowledged. First, as this was a retrospective observational study, missing data and variability in data quality were unavoidable. To address these issues, multiple imputation using chained equations was employed to enhance statistical efficiency. Second, the study’s sample size, especially the number of events, was lower than anticipated and may therefore compromise the precision of the calibration and discrimination estimate for model 2. Finally, our study was conducted at a single tertiary care centre in Northern Thailand. Although external validation demonstrated good reproducibility within our setting, further validation in lower-risk ASCVD populations and in multicentre setting is still be necessary to confirm generalisability across diverse settings.

## Conclusion

This external validation study confirms the predictive performance of CAC prediction. It demonstrates comparable discriminative performance to the development study without requiring updates. Although modest miscalibration was observed, in settings with a high CAC prevalence, the model effectively identified high-risk patients while maintaining consistent sensitivity and specificity. Further large-scale external validation studies are still required before widespread clinical implementation.

## Electronic supplementary material

Below is the link to the electronic supplementary material.

Supplementary Material 1

## Data Availability

All data generated or analysed during this study are included in this published article and its supplementary information files.
