# Supplementary material for: Performance of CAC-prob in predicting coronary artery calcium score: an external validation study in a high-CAC burden population
Source: BMC Med Inform Decis Mak. 2025 Aug 4;25:288. doi: 10.1186/s12911-025-03128-y (PMC12323013; doi:10.1186/s12911-025-03128-y)
Supplement: Supplementary file 1 — Supplementary Material 1 [file 12911_2025_3128_MOESM1_ESM.docx]

**Table S1.** Comparison of baseline characteristic between absence and presence of CAC score in validated sample

| **Prognostic factors** | **CAC=0**  **(n = 92)**  **n (%)** | **CAC= 1-99**  **(n = 86)**  **n (%)** | **CAC ≥100**  **(n=151)**  **n (%)** | **P value** |
| --- | --- | --- | --- | --- |
| Age (years) mean, SD | 57.4 ±11.3 | 63.6 ±8.8 | 69.2 ±9.4 | <0.001 |
| Male | 183 (49.7) | 28 (29.8) | 155 (56.6) | <0.001 |
| Smoking status |  |  |  | 0.002 |
| never smoking | 71 (77.2) | 70 (81.4) | 102 (67.6) |  |
| former smoking | 3 (3.3) | 2 (2.3) | 17 (11.3) |  |
| current smoking | 1 (1.1) | 3 (3.5) | 7 (4.6) |  |
| DM | 10 (10.9) | 15 (17.4) | 45 (29.8) | 0.001 |
| Hypertension | 38 (41.3) | 55 (64.0) | 112 (74.2) | <0.001 |
| Antihypertensive drug | 27 (29.4) | 47 (54.7) | 88 (58.3) | <0.001 |
| HDL-C(mg/dl) mean, SD | 58.2 ±14.2 | 55.8 ±15.3 | 55.0 ±23.1 | 0.057 |
| LDL-C(mg/dl) mean, SD | 124.8 ±47.7 | 116.3 ±41.1 | 104.4 ±43.9 | 0.006 |
| Triglyceride median, IQR | 108 (85, 126) | 114 (80, 131) | 111 (79, 128) | 0.832 |
| Dyslipidemia | 47 (51.1) | 57 (66.3) | 114 (75.5) | 0.001 |
| Lipid lowering drug | 26 (28.3) | 40 (46.5) | 74 (49.0) | 0.015 |
| eGFR mean, SD | 82.9 ±21.1 | 77.0 ±19.8 | 70.1 ±22.7 | <0.001 |
| Chronic kidney disease | 18 (19.6) | 31 (36.1) | 69 (45.7) | <0.001 |
| Symptomatic chest pain | 31 (33.7) | 32 (37.2) | 53 (35.1) | 0.616 |
| CAC for risk screening | 84 (91.3) | 76 (88.4) | 112 (74.2) | <0.001 |
| Thia CV risk median (IQR) |  |  |  | <0.001 |
| Low risk | 30 (32.6) | 27 (31.4) | 11 (7.3) |  |
| Intermediate risk | 15 (16.3) | 19 (22.1) | 35 (23.2) |  |
| High risk | 6 (6.5) | 18 (20.9) | 46 (30.5) |  |
| Pool cohort equation |  |  |  | <0.001 |
| Low -Borderline | 34 (37.0) | 20 (23.3) | 9 (6.0) |  |
| Intermediate risk | 12 (13.0) | 29 (33.7) | 37 (24.5) |  |
| High risk | 5 (5.4) | 15 (17.4) | 46 (30.5) |  |
| CAC score median, IQR | 0 (0, 0) | 23 (8.7, 61.4) | 816.6 (366.4,1509.2) |  |
|  |  |  |  |  |

**Abbreviations:** CAC, coronary artery calcium; CI, confidence interval; DM, diabetes mellitus; HDL-C, high-density lipoprotein cholesterol; IQR, interquartile range; LDL-C, Low-density lipoprotein cholesterol; SD, standard deviation

**Table S2** Risk classification table for each lower pairs cut point based on the estimated probability of CAC >0

| **Study** | **Cut point** | **number of classified patients** | **CAC =0** | **CAC 1-99** | **CAC ≥100** | **CAC ≥400** | **correct classified**  **rate** |
| --- | --- | --- | --- | --- | --- | --- | --- |
| Development | ≥0.50 | 203 | 45 (42%) | 69 (81%) | 89 (94%) | 36 (92%) | 78% |
|  | <0.50 | 84 | 62 (58%) | 16 (19%) | 6 (6%) | 3 (8%) |  |
| Validation | ≥0.50 | 241 | 38 (48%) | 67 (85%) | 136 (95%) | 101 (95%) | 81% |
|  | <0.50 | 60 | 41 (52%) | 12 (15%) | 7 (5%) | 5 (5%) |  |

*Total of 287 and 301 patients, based on unimputed data sets, were used for calculating the probability in development and validated dataset, respectively.

**Abbreviations:** CAC, coronary artery calcium.

**Table S3** Risk classification table for each cut point based on the estimated probability of CAC ≥100, when 0.50 was set as the lower pairs cut point to determine low risk of CAC >0

| **Study** | **Cut point** | **number of classified patients** | **CAC =0** | **CAC 1-99** | **CAC ≥100** | **CAC ≥400** | **correct classified**  **rate** |
| --- | --- | --- | --- | --- | --- | --- | --- |
| Development | ≥0.30 | 122 | 19 (35%) | 45 (62%) | 58 (64%) | 27 (75%) | 56% |
|  | <0.30 | 95 | 35 (65%) | 28 (38%) | 32 (36%) | 9 (25%) |  |
| Validation | ≥0.30 | 173 | 20 (53%) | 40 (60%) | 113 (83%) | 86 (85%) | 65% |
|  | <0.30 | 68 | 18 (47%) | 27 (40%) | 23 (17%) | 15 (15%) |  |

*Total of 217 and 241 patients, based on unimputed data sets, were used for calculating the probability in development and validated dataset, respectively.

**Abbreviations:** CAC, coronary artery calcium.

**Figure S1. Comparison of classification accuracy of CAC-prob for the lower and higher cut points between validation and development dataset.**

**
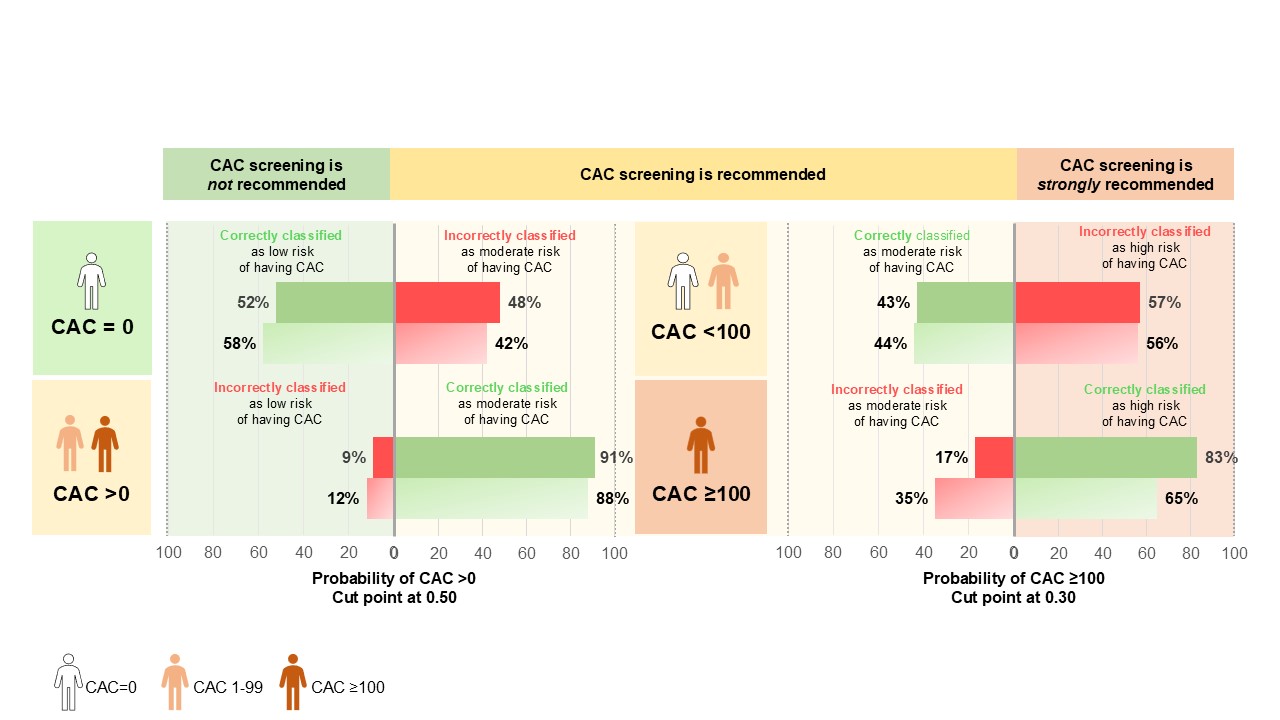
**

This figure illustrates the classification rate differences between the validation (solid colour) and development (faded colour) study. For CAC >0 probability, 3% more patients with CAC >0 were correctly classified as moderate risk (91% vs 88%), while 6% lower patients with CAC 0 were correctly classified as lower risk (52% vs 58%). For CAC >100 probability, 18% more patients with CAC ≥100 were correctly classified as high risk (83% vs 65%), while the correct classification rate for CAC <100 was only 1% lower than in the development study (43% vs 44%). **Abbreviation:** CAC, coronary artery calcium. Figure adapted from Wongyikul et al., 2024 [1].

**Reference**

1. Wongyikul P, Tantraworasin A, Suwannasom P, Srisuwan T, Wannasopha Y, Phinyo P. Prediction model for recommending coronary artery calcium score screening (CAC-prob) in cardiology outpatient units: A development study. PLoS One. 2024;19(9):e0308890. Published 2024 Sep 30. doi:10.1371/journal.pone.0308890
